# Utilizing fNIRS to investigate the impact of Baduanjin training on attentional function in post-stroke cognitive impairment patients: a study protocol for a randomized controlled trial

**DOI:** 10.1186/s12906-023-04284-2

**Published:** 2024-01-11

**Authors:** Xingchen Zhou, Yiwen Wan, Zhengxian Xu, Cancan Yu, Ziyi Wu, Zesen Zhuang, Rui Xia, Hongyu Wang, Shangjie Chen

**Affiliations:** 1 Graduate School, Bengbu Medical College, Bengbu, 233000 Anhui China; 2 Department of Rehabilitation, The People’s Hospital of Baoan Shenzhen, Bengbu Medical College, Bengbu, 233000 China; 3 Shunde Women and Children’s Hospital of Guangdong Medical University(Maternity &, Child Healthcare Hospital of Shunde Foshan), Foshan, China

**Keywords:** Baduanjin, Post-stroke cognitive impairment, Attentional lateralization, Randomized controlled trial

## Abstract

**Background:**

Post-stroke cognitive impairment (PSCI) is a prevalent complication among stroke survivors. It not only directly impacts patients' cognitive abilities but also hampers their capacity to regain independence in daily activities, consequently diminishing their quality of life. Among the various cognitive deficits following stroke, impaired attention is the most frequently observed, influencing not only daily functioning but also higher cognitive functions like working memory, executive functioning, and language.Emerging evidence indicates that Baduanjin, a traditional Chinese exercise, may have a positive impact on enhancing attention in older adults with mild cognitive impairment and stroke survivors. However, the precise mechanisms behind this effect remain unclear. In this study, we employed Baduanjin training as an intervention to address attention decline in post-stroke cognitive impairment patients and to delve into the potential mechanisms through which Baduanjin training may enhance attention in individuals with PSCI.

**Methods:**

In this prospective randomized controlled trial, we plan to recruit 72 participants diagnosed with post-stroke cognitive impairment (PSCI). These participants will be randomly assigned in a 1:1:1 ratio to one of three groups: Baduanjin training(left hemisphere stroke and right hemisphere stroke) and conventional treatment.The conventional treatment group will receive standard rehabilitation sessions. In addition to conventional treatment, participants in the octogenarian training groups will undergo octogenarian training sessions lasting 40 min, five times a week, over a total period of 12 weeks.The primary outcome measures will include the Montreal Cognitive Assessment (MoCA) scale and the Attentional Lateralization Index. These assessments will be conducted by a trained evaluator before the start of the intervention and at weeks 6 and 12 after the intervention begins.Secondary outcome measures will be assessed using the baseline Mandarin version of the Oxford Cognitive Screening (OCS-P) scale, the simplified Fugl-Meyer Motor Function Assessment (FMA) scale, the Pittsburgh Rehabilitation Participation (PRPS) scale, and the Activities of Daily Living (ADL) scale before and after the intervention, respectively.

**Discussion:**

This trial aims to examine the impact of Baduanjin training on attentional lateralization among patients with post-stroke cognitive impairment (PSCI). Functional brain imaging utilizing near-infrared spectroscopy will be employed to investigate how Baduanjin exercise influences the structural and functional connectivity of distinct brain regions or brain networks.

**Trial registration:**

Chictr.org.cn, ID: ChiCTR2300076533. Registered on 11 October 2023.

## Introduction

### Background

Post-stroke cognitive impairment (PSCI) is a prevalent complication among stroke survivors, with approximately 50% of patients experiencing cognitive impairment in the early post-stroke phase [1]. Alarmingly, up to 32% of patients continue to suffer from cognitive impairment even three years after their initial stroke [2]. PSCI not only directly impacts patients' cognitive abilities [3] but also hinders their capacity to perceive and adapt to their external environment. It significantly disrupts the recovery of their daily living skills and diminishes their overall quality of life [4]. Moreover, the challenges in adhering to therapeutic instructions often have a profound impact on the efficacy and progress of rehabilitation training, further impeding functional recovery [5].

Impaired attention is one of the most common cognitive deficits following a stroke, with approximately 46% to 92% of patients experiencing attentional deficits in the acute phase [6]. These deficits persist in 20% to 50% of cases for several years post-injury [7, 8]. The attentional system comprises three functionally and structurally independent subsystems: vigilance, orienting, and executive control [9].Vigilance refers to the state of heightened sensitivity to external information and its maintenance. Orientation involves the selection of specific information from a vast amount of external input. On the other hand, executive control functions encompass the ability to execute complex behaviors, monitor, and resolve conflicts [10]. These three attentional subsystems are governed and processed by three distinct brain networks: the dorsal attention network (DAN), the ventral attention network (VAN), and the executive control network (ECN). These networks, while relatively independent, work in tandem to complete the attentional process and achieve cognitive control [11]. Our current study suggests that attention processing in the two hemispheres exhibits asymmetry, leading to a biased lateralization of the attentional network. Specifically, there is no significant lateralization for the vigilance function [12, 13]. However, significant left visual field-right hemisphere lateralization is observed for the subnetworks involved in orientation and executive control [14, 15]. Imaging studies have also revealed a strong right lateralization of the VAN in conjunction with the ECN, while the DAN exhibits a symmetrical distribution in both cerebral hemispheres. This implies that the attentional network displays a right-sided lateralization balanced with bilateral coordination, enhancing the efficiency of attentional processes while maintaining bilateral coordination.Nonetheless, it is this right-sided lateralization of attention that leads to the clinical prevalence and severity of left-sided attentional impairments resulting from right brain injuries [16]. Stroke patients experience attentional dysfunction due to damage to the dominant side of the brain region, disrupting the lateralization pattern of the attentional network. Previous studies have demonstrated that right hemispheric infarcts result in an advantage in left hemispheric orienting function processing, while left hemispheric infarcts lead to the loss of this advantage and impair orienting function [17]. Thus, the lateralization pattern of the attentional network in stroke patients is a crucial factor contributing to attentional dysfunction.

Baduanjin is a traditional Chinese health exercise, which is widely spread because of the advantages of soothing, simple, and high safety [18]. In recent years, Baduanjin exercises have found extensive application in stroke rehabilitation, demonstrating significant efficacy in primary stroke prevention, post-stroke motor function improvement, daily living activities, quality of life enhancement, balance function enhancement, and cognitive function improvement [19, 20]. A study involving 41 participants directly illustrated that six months of Baduanjin training led to improvements in overall cognitive function, executive function, and memory in patients with post-stroke cognitive impairment (PSCI). It also reduced alert reaction times and improved attention in these patients [21]. Other studies have indicated that Baduanjin, either alone or in conjunction with herbal or acupuncture therapies, can enhance attentional functions such as concentration in PSCI patients [22–24]. These findings suggest that Baduanjin has a positive impact on attention in individuals with PSCI, although the exact underlying mechanism remains to be elucidated.

Functional near-infrared spectroscopy (fNIRS) is a non-invasive brain imaging technique that relies on the varying absorption of near-infrared light within the 600–900 nm wavelength range by oxyhemoglobin and deoxyhemoglobin in brain tissue. This allows for real-time and direct detection of cortical oxygenation activity [25]. The relatively low cost of fNIRS, minimal requirements for testing environment and subject mobility, and few contraindications have led to a growing number of researchers utilizing fNIRS in stroke-related studies [26]. To investigate the effects and underlying mechanisms of 12 weeks of Baduanjin training on attentional lateralization and attentional networks in patients with post-stroke cognitive impairment (PSCI), we propose a prospective, randomized controlled trial (RCT). The primary goal of this trial is to assess the impact of octogenarian training on attentional lateralization in individuals with PSCI. Additionally, we seek to explore how Baduanjin exercise influences the structural and functional connectivity of specific brain regions and brain networks using near-infrared spectroscopic functional brain imaging.

## Methods

### Study design

**Fig. 1:**
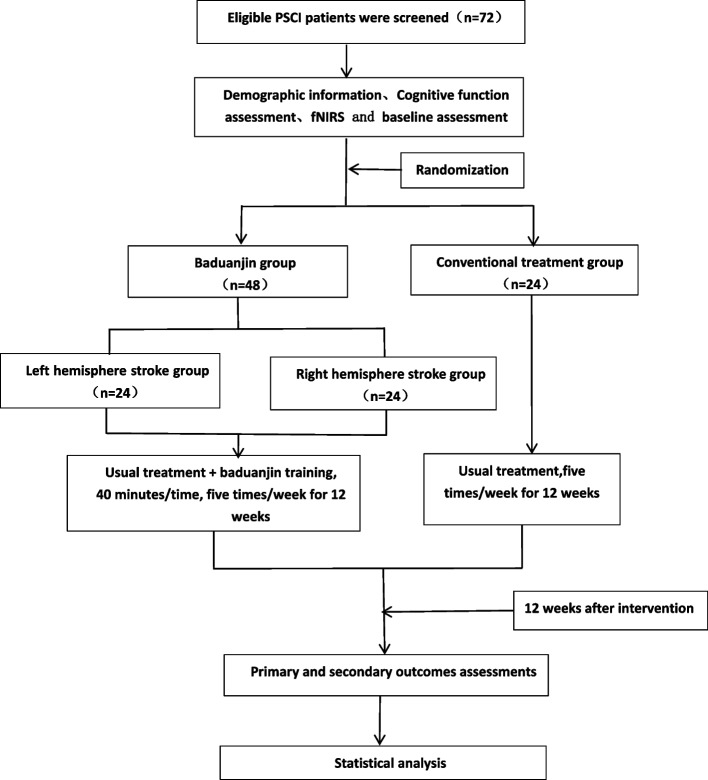
Flow chart of the trial

**Fig. 2:**
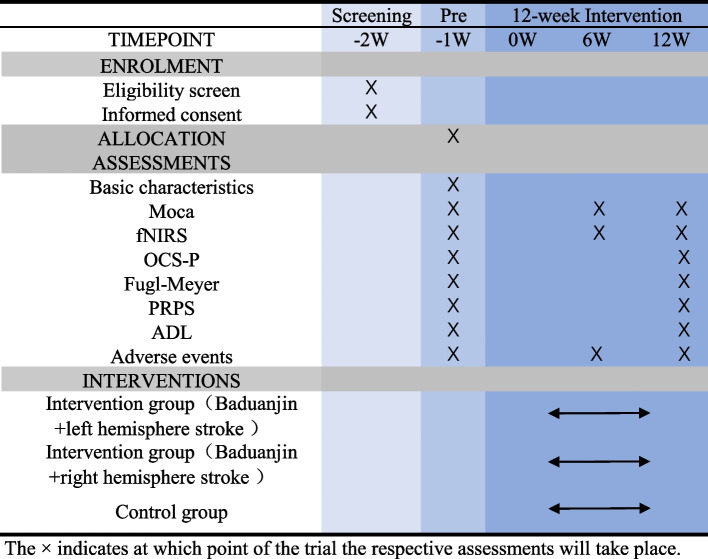
Schedule of enrolment, assessments, and interventions

The study design employed in this research is a randomized controlled trial, incorporating allocation concealment and assessor blinding. A total of 72 eligible participants will be randomly allocated to either the 12-week Baduanjin exercise intervention (with sessions lasting 40 min, conducted five times a week) or the 12-week regular activity control group.In addition to the Attentional Lateralization Index (ALI) and Overall Cognitive Functioning (OCF), which will be assessed by a professional evaluator using the Montreal Cognitive Assessment (MoCA) scale before the intervention and at the onset of the intervention (at weeks 6 and 12), cognitive dimensions will be evaluated using the Oxford Cognitive Screening (OCS-P) scale, motor function will be assessed using the Fugl-Meyer scale, participation level will be rated using the Pittsburgh Rehabilitation Participation Scale (PRPS), and activities of daily living will be measured. These assessments will be conducted at baseline and at the end of the 12-week intervention. For a comprehensive understanding of the study procedures and the timeline for the evaluation of the results, please refer to Figs. 1 and 2.

### Inclusion criteria

(1) Met the diagnostic criteria for stroke, as outlined in the "Diagnostic Points for Various Major Cerebrovascular Diseases in China 2019," revised by the Expert Committee on Guideline Consensus of the Cerebrovascular Group of the Chinese Medical Association Neurology Section and the Chinese Medical Association Neurology Sect. (2) Diagnosed with mild cognitive impairment according to the criteria for "mild vascular neurocognitive disorder" as specified in the Diagnostic and Statistical Manual of Mental Disorders, 5th edition (DSM-5).(3) Experienced their first stroke with a disease duration of ≤ 1 year.(4) Aged between 45 and 75 years old.

(5) Maintained a stable condition with conscious and stable vital signs.(6) Demonstrated the ability to walk independently and safely for a distance of 10 m or more without the need for physical assistance or assistive devices.(7) Possessed the use of their dominant hand (right hand).(8) Provided informed consent and participated voluntarily in the study.

### Exclusion criteria

(1) Severe speech, visual, hearing impairments, or mental disorders that interfere with cognitive examinations.(2) Presence of other medical conditions known to cause cognitive dysfunction, such as brain tumors, traumatic brain injuries, or cerebral parasitosis, as confirmed through medical examinations.(3) Pre-existing conditions related to cognitive impairment or the use of medications targeting cognitive impairment prior to the onset of the condition, as verified by prior medical records or confirmed by a clinician or family member.(4) Beck Depression Inventory 2nd Edition (BDI-II) scores exceeding 13 points, indicating significant depressive symptoms.(5) A history of alcohol or drug abuse.(6) Concurrent serious medical conditions affecting the heart, liver, kidneys, endocrine system, or hematopoietic system.(7) Participation in other clinical trials that could influence the evaluation of this study's results.Participants meeting any of these exclusion criteria were not included in the study.

### Recruitment

This study aims to enroll stroke patients from the People's Hospital of Bao'an District, Shenzhen City, Guangdong Province, as well as its affiliated community health centers, including Dalang and Xingdong. Recruitment will be carried out by trained medical personnel who will assess eligibility based on the inclusion and exclusion criteria.Before conducting the baseline assessment, a research assistant will obtain written informed consent from individuals who express their willingness to participate in the study.

### Randomization, allocation concealment, and blinding

After the baseline assessment, eligible participants will be randomly allocated to either the Baduanjin exercise group or the routine activity control group using a randomization process. A random number grouping table will be generated based on the assigned number of cases and a random proportion. These randomization tables will be created using SAS 9.0 and managed by independent research assistants who are not involved in participant recruitment, assessment, or intervention.Independent research assistants will notify eligible participants of their assignment results via phone communication. Due to the differing forms of intervention, it is not feasible to maintain blinding for researchers, participants, and coaches throughout the experiment. However, laboratory technicians and researchers responsible for statistical analyses will remain blinded.

### Sample size

The Montreal Cognitive Assessment (MoCA) scale served as the primary outcome measure for the final evaluation. Based on the results of a literature search, the effect size for both study groups was determined to be 0.923502 using Gpower3.1.9.2 software [21]. To achieve a test power of 0.80, a total sample size of 60 participants was calculated, with 20 participants in each group. Accounting for a potential dropout rate of 20%, the overall sample size required for this study was determined to be 72 participants, evenly distributed with 24 participants in each group.

### fNIRS data collection

To investigate the potential mechanisms of Baduanjin training on post-stroke cognitive dysfunction, this study will collect functional near-infrared spectroscopy (fNIRS) data from participants both before and after 12 weeks of intervention. The equipment utilized for this purpose is the Wuhan Zilian Hongkang model BS-3000 fNIRS equipment.Prior to data collection, the study's objectives were thoroughly explained to the participants. They were instructed to keep their heads at rest during the tests and minimize swallowing movements to minimize potential testing errors. The areas sampled include the premotor cortex (PMC), supplementary motor area (SMA), primary motor cortex (M1), and sensorimotor cortex (SMC).The 37-channel fiber optic cap employed in this study comprises 12 light sources and 12 detectors, spaced 3 cm apart in a rectangular grid of 3 rows by 8 columns. Light is emitted from a source and penetrates the scalp and skull, where it is absorbed by oxyhemoglobin and deoxyhemoglobin in the cortex. The remaining light is then detected. Changes in hemoglobin concentration within the region traversed by the channel serve as indicators of local neuronal activity.

### Attention to lateralization tests

Attention is a fundamental cognitive function, and the exploration of its characteristics and processes is of paramount importance. The Attention Network Test (ANT) is a valuable tool designed to provide comprehensive scores assessing the efficiency of alerting, orienting, and executive control. In this study, the lateralized attention network test (LANT) was utilized, and the test was conducted using the E-prime 2.0 software.During the LANT, target arrows were presented on a computer screen, with the subject positioned at a distance of approximately 60 cm from the screen. A total of 318 trials were administered, which included 30 practice trials and 288 formal trials, organized into 4 phases. Participants had the opportunity to rest after completing each phase, resulting in a total test duration of approximately 30 min. Assessments were conducted both before the intervention and at the twelfth week of the intervention.

## Intervention

### Conventional treatment group

The foundational treatment was carried out in accordance with the 2017 Expert Consensus on the Management of Cognitive Impairment after Stroke. The specific treatment plans were devised by the attending physicians at the respective hospitals where the patients were receiving care. The personnel involved in this research group did not actively partake in the treatment procedures. Instead, they objectively documented the treatment processes throughout the study duration.Comprehensive records of patients' daily treatment and medication were meticulously maintained during the 12-week intervention period.

### Baduanjin group

In addition to regular treatment, we incorporated Baduanjin training as a supplementary intervention. This training followed the standards outlined in the "Fitness Qigong—Baduanjin" guidelines issued by the State General Administration of Sports in 2003. It consists of ten movements, including the preparatory posture, holding the sky with both hands to manage the three jiao, opening the bow left and right as if shooting an eagle, regulating the spleen and stomach to be lifted single-handedly, looking backward for the five labors and seven injuries, wagging the head and tail to remove the fire in the heart, climbing on the feet with both hands to strengthen the kidney and waist, saving the fist to increase the qi strength of the eyes in anger, eliminating diseases by focusing on the back's seven bumps, and concluding with the closing posture.A trained therapist was present during the training sessions to provide guidance and ensure the safety of participants. The Baduanjin training occurred five times a week, with each session lasting 40 min, and continued for 12 weeks.To eliminate the potential influence of excessive exercise, all participants in both the Baduanjin exercise group and the conventional rehabilitation treatment control group were advised to refrain from engaging in any other forms of exercise during the trial. Their adherence to the Baduanjin exercise methodology was rigorously recorded throughout the study period.

### Assessment of results

The variables considered in this trial encompass basic participant information, primary, and secondary outcomes. Baseline characteristics will be assessed within the window of 1–2 weeks before random assignment.The primary outcomes, namely Overall Cognitive Functioning and Attentional Lateralization Index, will be measured at three time points: pre-intervention, week 6, and week 12 of the intervention.Secondary outcomes will be evaluated both before and after the intervention period. All assessments of primary and secondary outcomes will be conducted by experienced staff at the Shenzhen Bao'an District People's Hospital. These assessors will be blinded to the participants' allocation, ensuring the integrity of the study.

### Basic information

Recruiters will gather comprehensive demographic information from participants, including age, gender, ethnicity, occupation, education level, marital status, past medical history, family medical history, dietary habits, alcohol consumption, frequency of exercise, and more.In addition, prevalence data, such as the duration of stroke and the site of the lesion, as well as the presence of hemiparesis, will also be collected using a self-designed questionnaire. The degree of neurological impairment will be evaluated using the National Institutes of Health Stroke Scale (NIHSS).All baseline measurements will be conducted before participants are randomized into their respective groups.

## Primary outcomes

### Global cognitive function

Global cognitive functioning will be evaluated using the Montreal Cognitive Assessment Scale (MoCA). This scale is widely recognized for its validity and reliability in assessing overall cognition in individuals with mild to moderate stroke [27]. The MoCA assesses various cognitive domains, including attention and concentration, executive functioning, memory, language, visual-spatial skills, abstract thinking, computation, and orientation. Completing the MoCA examination typically takes approximately 10 min. The total score on the MoCA ranges from 0 to 30, with lower scores indicating poorer cognitive function. Additionally, one point is added to the final score for individuals with 12 or fewer years of education.

### Attention to the lateralization index

The study assessed alerting efficiency, localization efficiency, and executive control effects as part of the attentional network effects. These effects were separately calculated for the left and right visual fields.Alerting network effect was determined by subtracting the time (or error rate) of the no-cue response from the time (or error rate) of the dual-cue response.Localization efficiency was calculated by subtracting the time (or error rate) of the dual-cue response from the time (or error rate) of the valid cue response.Executive control effect was derived by subtracting the time (or error rate) of the response in the inconsistent direction of the arrows condition from the time (or error rate).It's important to note that higher values indicated greater vigilance and orientation effects, while lower values indicated stronger executive control effects [12].

### Secondary outcome indicators

#### Rating of cognitive dimensions

Cognitive dimensions will be evaluated using the Mandarin version of the Oxford Cognitive Screening Scale (OCS-P). This scale assesses five cognitive domains, which include memory, verbal cognition, numerical skills, practical abilities, and attentional and executive functions. The OCS-P serves as a valuable assessment tool for the rapid screening of cognitive function in stroke patients, providing guidance for comprehensive cognitive assessments [28].

### Motor function assessment

The Fugl-Meyer Motor Function Assessment Scale (FMA) was employed to evaluate the motor function of the study participants. The FMA is widely regarded as the gold standard for assessing motor function in individuals with hemiplegia following a stroke. This comprehensive assessment encompasses the measurement of voluntary movement, speed, coordination, and reflex activity. As such, it serves as one of the primary components of the secondary outcome measures [29].

### Participation level assessment

The level of participation was assessed using The Pittsburgh Rehabilitation Participation Scale (PRPS), a six-point scale with the following ratings:Score of 1 (none): The patient refused to participate in the entire training or did not engage in any of the exercises within the training.

Score of 2 (poor): The patient refused to participate in at least half of the training or did not take part in at least half of the exercises.Score of 3 (fair): The patient participated in almost all of the exercises but did not exert maximal effort, did not complete most of the exercises, or required significant encouragement.Score of 4 (good): The patient participated to the best of their ability, completing as many exercises as possible but not all, and followed instructions rather than showing intrinsic interest in the exercises and future treatment.Score of 5 (very good): The patient participated in and completed all exercises to the best of their ability but required instruction rather than displaying a strong interest in the training and future treatment.

Score of 6 (excellent): The patient participated in and completed all exercises to the best of their ability and demonstrated a strong interest in the training and future treatment.

The PRPS is recognized for its validity and reliability, providing a quantitative reflection of a patient's rehabilitation participation or initiative. It serves as a credible and practical rehabilitation participation scale [30].

### Activities of Daily Living (ADL)

The assessment of the ability to perform activities of daily living (ADL) will be conducted using the Barthel Index (BI). This standardized scale is widely employed to evaluate functional disability in ADLs. The BI comprises 10 items, and the total score ranges from 0 to 100, with higher scores indicating a better ability to independently manage daily life tasks.The BI is known for its comprehensive, accurate, and efficient assessment of an individual's capacity to carry out activities of daily living [31].

### fNIRS data analysis methods

#### Pre-processing

The data processing steps for functional near-infrared spectroscopy (fNIRS) were as follows:

①The fNIRS data was initially converted from NIRS format to MAT format.②Wavelet-MDL (Minimum Description Length) was employed to remove noise from sources such as heartbeat and breathing.③An HRF (Hemodynamic Response Function) low-pass filter was applied to eliminate signal drift and artifacts.④The necessary parameters for the General Linear Model (GLM) were selected, and β-values were estimated for the model using a t-test with a significance threshold of *P* < 0.05.To account for multiple comparisons, the False Discovery Rate (FDR) correction was utilized. This correction method was employed to output statistical information, including T-values, activation regions, and statistical threshold values.

#### Functional connectivity analysis of the attentional network

The degree of signal synchronization in the time domain during the initial 6-min resting phase was assessed using Pearson correlation analysis across all measured brain regions. This analysis provided a connectivity matrix that captured both intra- and interhemispheric connectivity relationships. The elements within the matrix represented the correlation coefficients of the paired channels, with rows and columns corresponding to the channel numbers.Graph Theoretical Analysis was subsequently employed to calculate various node metrics. Nodes with a sparsity greater than 20% were selected for analysis to assess the overall characteristics of the brain network organization. The analysis was conducted using the Graph Theoretical Network Analysis (GRETNA) toolbox. The metrics calculated included global efficiency (reflecting network efficiency), local efficiency (reflecting local network efficiency), and the small-world network property. Higher values of these metrics indicate higher network efficiency and adherence to a small-world network structure.

### Safety measurements

The assessment of safety in this study focused on monitoring the incidence and severity of adverse events. Any adverse events occurring during the study were diligently documented in the Adverse Event Record Sheet, which included details such as symptoms, onset time, duration, and any treatment measures administered.Adverse events were categorized into three levels based on severity:general adverse events,significant adverse events and serious adverse events.For serious adverse events, a dedicated report form was completed. Safety evaluation was conducted by synthesizing all relevant factors. The evaluation parameters included:Level 1: Safe, without any adverse reactions;Level 2: Relatively safe, with mild adverse reactions that did not require treatment, allowing for continued training;Level 3: Safety concerns, with a moderate degree of adverse reactions that could continue with treatment;Level 4: Due to serious adverse reactions, discontinuation of the study was necessary.Depending on the circumstances, safety assessments were performed for subjects at the conclusion of the intervention to ensure their well-being throughout the trial.

### Research process and data management

For the randomization process, we will utilize the Clinical Trial Public Management Platform known as ResMan, which is part of the China Clinical Trial Registry. Subjects will be assigned equally to each group at a 1:1:1 ratio.Furthermore, the research data generated during the study will be made publicly available and disseminated through this platform upon the conclusion of the study.

### statistical analysis

The data in this study were subjected to statistical analysis using SPSS 26.0 software. All statistical tests employed a two-sided approach with a significance level (α) set at 0.05. Results were considered statistically significant when the *P*-value was ≤ 0.05 and highly significant when the *P*-value was ≤ 0.01.Count data were described using frequencies, percentages, or component ratios. Measurement data were described using mean ± standard deviation or median described.One-way ANOVA was used for data satisfying normal distribution, rank sum test for those not satisfying normal distribution, and chi-square test for count data.Multiple samples were analyzed by ANOVA, variance chi-square test for two-by-two comparisons, and LSD table for chi-square and Games-Howell table for non-chi-square.Mixed linear models were used to analyze the relationship between time and effect of interventions on cognitive function.

### Ethical issues

This study protocol strictly adheres to the principles outlined in the Declaration of Helsinki. It has undergone thorough review and received approval from the Ethics Committee of Shenzhen Bao'an District People's Hospital(Ethics Approval No. BYL20230815).Prior to participating in the study, all participants will engage in comprehensive discussions regarding the potential benefits and risks. They will provide voluntary, written informed consent to ensure full awareness and understanding of their involvement in the study.

### Dissemination

The study protocol has been registered and can be accessed through the Chinese Registration website.(registered on ChiCTR.org with the identifier ChiCTR2300076533). The results of this study will be presented at local, national, or international conferences on cognitive rehabilitation and will be submitted as a manuscript to a peer-reviewed journal. The main findings of this study will also be shared with all participants and disseminated through courses, presentations, and the Internet to researchers, health service providers, health care professionals, and the general public, regardless of the level or direction of impact.

## Discussion

Baduanjin intervention method is not only safe and effective but also convenient and easily accessible, making it suitable for a broad spectrum of patients with Post-Stroke Cognitive Impairment (PSCI).Numerous studies have illustrated the positive impact of Baduanjin on PSCI-related attention issues [32], along with its potential to modulate the pattern of attentional lateralization [33–35]. However, existing studies have provided only a cursory overall assessment without a systematic and in-depth evaluation of how Baduanjin specifically affects the various subsystems of the attentional network in PSCI.The primary objective of this study is to delve into the precise effects of Baduanjin training on the subsystems of the attentional network. We aim to determine whether Baduanjin training can effectively address the issue of reduced attention in PSCI patients by influencing the pattern of attentional lateralization and consequently improving their attention.

The trial will employ a rigorous randomized allocation method, ensuring that assessors and statistical analysts are kept unaware of the group assignments to minimize bias. To ensure participants acquire a mastery of standardized and precise Baduanjin movements, qualified physical activity instructors will serve as Baduanjin exercise coaches. This approach is expected to yield highly reliable results.Increasing the frequency of Baduanjin training to five sessions per week and shortening the intervention duration to 12 weeks is designed to facilitate clinical adoption and patient adherence after hospital discharge. This approach is both practical for scientific research and aligns with the principles of Chinese medicine rehabilitation intervention in PSCI.Furthermore, to mitigate the influence of factors such as stroke location on the results of imaging data analysis, this study innovatively utilizes fNIRS-based analysis of the lateralization index to conduct an in-depth exploration of the impact of Baduanjin on the lateralization of the attentional network. The findings from this study are expected to contribute significantly to improving attention in patients with PSCI and may have broader applications for the elderly population in community-based settings.

In summary, this study represents the first randomized controlled trial aimed at investigating the impact of octopus training on attention in patients with PSCI by examining alterations in the pattern of attentional network lateralization. If the study is successfully completed and yields meaningful results, it has the potential to provide a scientific foundation for implementing Baduanjin interventions to improve attention in elderly PSCI patients. Moreover, it may offer initial insights into the mechanisms underlying the beneficial effects of Baduanjin on PSCI rehabilitation. These findings could facilitate the clinical adoption of Baduanjin interventions and promote sustained patient engagement with the practice after hospital discharge.

## Data Availability

Not applicable. No data have been generated. Raw data sharing will be available after 1 March. 2025 by contacting the corresponding author.
